# The effect of interdisciplinary treatment on sickness absence and disability pension among chronic pain patients on partial disability pension

**DOI:** 10.1371/journal.pone.0317797

**Published:** 2025-02-04

**Authors:** Lea Constan, Paolo Frumento, Riccardo LoMartire

**Affiliations:** 1 Independent Researcher, Sweden; 2 Department of Political Sciences, University of Pisa, Pisa, Italy; 3 Center for Clinical Research Dalarna – Uppsala University, Falun, Region Dalarna, Sweden; 4 School of Health and Welfare, Dalarna University, Falun, Sweden; Center for Research and Technology Transfer, VIETNAM

## Abstract

Studies evaluating the effects of interdisciplinary treatment (IDT) on sickness absence and disability pension (SA/DP) have yielded contradictory findings. Evidence indicates that positive treatment effects are restricted to patients with a poor SA/DP prognosis. This study therefore analyzed the effect of IDT in working age patients on partial disability pension, which is a group with a particularly poor prognosis. With data from 479 patients on partial disability pension, this register-based cohort study compared the effects of IDT to those of unspecified interventions. We considered two response variables: total net SA/DP days across the span of three years from the first visit to a Swedish specialist pain clinic, and the risk of having the maximum possible 1096 SA/DP days over the same period. Our results showed that both the total net SA/DP days (mean difference: 11; 95% confidence interval: -30 to 51) and the risk of 1096 SA/DP days (risk ratio: 1.0; 95% confidence interval: 0.6 to 1.4) were similar irrespective of intervention type. Under our theoretical model, we thereby found no support in favor of IDT over less intensive interventions in working age patients with partial DP. This raises questions about the specific criteria under which IDT proves effective.

## Introduction

Chronic pain is a prevalent condition globally that imposes a significant burden on society through sickness absence and disability pension (SA/DP) [1–3]. Consequently, SA/DP reduction is a public health priority [4]. Stemming from the biopsychosocial theory of pain, it is today conventionally understood that the interdisciplinary treatment (IDT), a widespread chronic pain core intervention that adapts multiple treatment modalities to the patient’s needs, is the best approach to improve the situation of chronic pain patients [2]. In Sweden, a 2009 government policy was implemented to nationally fund IDT [5]. However, studies evaluating the effectiveness of IDT in reducing SA/DP have reported mixed findings, ranging from positive to negative effects compared to those of less intensive treatments [6–9]. The large variation in patient characteristics and in IDT program configurations complicate the evaluation process [10–12]. Despite this, previous results have suggested that treatment effectiveness could depend on a threshold effect where a positive outcome is dependent on a poor SA/DP prognosis [13,14]. Indeed, we recently found that IDT reduced the risk of maximum SA/DP over a three-year period relative to less intensive interventions [15]. In the Swedish health system, a subpopulation which could correspond to this criterion is composed by the recipients of partial disability pension. This study compared net SA/DP days between chronic pain patients on partial disability pension receiving either IDT or unspecified interventions under the hypothesis that IDT would decrease SA/DP more than the unspecified interventions.

## Materials and methods

### Design

This register-based cohort study examined the net SA/DP days between patients in either IDT programs or unspecified interventions from three years before to three years after Swedish specialist healthcare entry. The total SA/DP days during the final three years were compared across the two treatment types. The sampling frame was the Swedish Quality Registry for Pain Rehabilitation on the 1^st^ of June 2018, which includes patients with chronic pain conditions that are characterized by physical, emotional, and social impairments [16]. Patients with chronic pain for at least 90 days, aged between 30–50 years, on partial disability pension, and who had visited a specialist clinic with an annual volume of at least 20 patients for both IDT programs and unspecified treatments in the period 2011-01-01 to 2015-12-31 were included. Patients with a registered ICD-10 neoplasm diagnosis (C00-D49) in the previous five years, zero or full disability pension at baseline, or a visit to an IDT specialist clinic in the previous two years were excluded. Data on SA/DP were obtained from the Swedish Social Insurance Agency on the 1^st^ of February 2019, sociodemographic information was collected from Statistics Sweden on the 1^st^ of March 2019, and data on ICD-10 neoplasm diagnoses were sourced from the National Board of Health and Welfare on the 1^st^ of February 2020 [17–19]. This study was approved by Uppsala’s Medical Research Ethics Committee (DNR 2018/036) and a written informed consent was obtained from all patients. The authors had no access to information that could identify individual patients. The Supplementary Materials contain study details: the patient selection procedure in S1 Fig; our conceptual model in S2 Fig; the confounder distribution in S3 and S4 Figs; the outcome distribution in S5 Fig; missing data in S6 Fig; analytical details in S1–S3 Tables and S7 Fig.

### Interdisciplinary treatment and unspecified interventions

IDT is provided by around 40 specialist healthcare clinics across Sweden, which report their data to the Swedish Quality Register for Pain Rehabilitation [12]. National guidelines indicate that experienced interprofessional teams administer IDT in cohesive programs that are tailored to each patient’s needs [20]. However, in practice, IDT programs may differ between clinics and primarily consist of group-based activities like cognitive behavioral therapy, physical therapy, and occupational training. These activities are carried out by a team of healthcare professionals, including physicians, physiotherapists, occupational therapists, and social workers [12,20]. While individual patient information regarding IDT programs is not recorded, the programs generally last between 4 and 12 weeks and include 30 to 100 hours of therapy [12]. These hours are usually delivered at a frequency of 2 to 5 days per week. Not all patients visiting specialist clinics receive IDT programs. Instead, some patients may be offered other interventions or recommendations following team evaluation, depending on factors such as patient preferences, healthcare resources, and other unknown factors. These unspecified interventions vary in intensity but are generally less comprehensive than IDT programs.

### Social insurance

The Swedish social insurance system provides economic stability in case of work incapacity for all Swedish residents aged 16 to 64 with minimum income from employment or unemployment. SA is possible from age 16 and can be granted as full or part time (25%, 50%, or 75%) of ordinary work hours, with episodes generally compensated for by the employer during the first 14 days [18]. Episodes are reimbursed and recorded by the Swedish Social Insurance Agency from the 15^th^ day. DP can be granted to individuals whose working capacity is impaired for the foreseeable future. Although it is possible to terminate DP, it is rare; instead, the prevalence of DP is increasing with age. DP is granted either full or part-time, and is recorded by the Swedish Social Insurance Agency from the first day [18]. Patients on partial DP are also eligible for SA up to full time of ordinary working hours. In this study, we combined net days of registered SA and DP to create a composite measure of SA/DP, where partial days were summed into full days (e.g., two days with 50% SA/DP were summed to one full day with SA/DP).

### Conceptual model of intervention group equivalence

Observational studies that evaluate treatment effectiveness may be biased if the intervention groups are not equivalent at baseline. To address this issue, it is recommended that confounders are adjusted for in the analysis [21]. We identified important confounders in the scientific literature that likely influenced both participation in an IDT program and the reception of SA/DP under the domains of SA/DP history, policy, sociodemographics, and disability (see S2 Fig). Noteworthy is that we adjusted for country of birth in the analyses in place of ethnicity, because such data not is available in the Swedish public registers. The motivation behind our conceptual model is presented elsewhere [15].

### Statistical analysis

To provide a descriptive overview, we visualized the daily trend in mean net SA/DP days per 1000 patients from three years before to three years after specialist healthcare. To compare the total net SA/DP days over the three-year period (1096 days) following specialist healthcare entry between intervention groups, we used an ordinal logistic regression model on complete cases (rms 6.7-1 in R v4.3.0), adjusted for the theory-driven confounders previously described (S2 Fig and S1 Table) [22]. In specific, we predicted the SA/DP for all patients set at both IDT and unspecified interventions using the fitted model, and then calculated the mean difference in total SA/DP days from the marginal distributions [23,24]. A linear relationship was assumed for continuous confounders, while categorical confounders were included as dummy-coded factors (S2–S3 Tables). Finally, we also compared the marginal risk of having the maximum possible 1096 SA/DP days between the intervention groups, derived from a logistic regression model configurated as our primary model (R v4.3.0). Inference was based on empirical bootstrap with 10,000 replicates.

## Results

In total, 479 (1.5%) of the 31,707 patients that visited a Swedish specialist clinic in the period 2011 to 2015 met our eligibility criteria. Approximately half of them were included in an IDT program (*n* = 223) and the other half in an unspecified intervention (*n* = 256). Noteworthy was that IDT-admitted patients were more educated, had a higher degree of employment, a higher confidence in recovery, less SA in the year before specialist healthcare entry, and a lower extent of partial disability pension at start (Table 1; S3 and S4 Figs). Whereas data on SA/DP were complete for all patients, 15 (6.7%) of the IDT patients and 24 (9.4%) of the patients in an unspecified intervention had not reported their confidence in recovery or their degree of pain interference.

**Table 1:** Sample baseline characteristics.

	Interdisciplinary treatment (*n = 223*)	Unspecified intervention (*n* = 256)
Sociodemographics		
Age (years)^a^	44 (5)	43 (5)
Female^b^	182 (82)	197 (77)
Geographical region^b^		
Stockholm	42 (19)	26 (10)
Southern Sweden	78 (35)	85 (33)
Southwestern Sweden	36 (16)	59 (23)
Central Sweden	42 (19)	67 (26)
Northern Sweden	25 (11)	19 (7)
Country of birth^b^		
Sweden	197 (88)	223 (87)
Other European country	19 (9)	19 (7)
Non-European country	7 (3)	14 (5)
Education (years)^b^		
Elementary (<10)	20 (9)	42 (16)
High school (10–12)	150 (67)	165 (64)
University/college (>12)	53 (24)	49 (19)
Employed^b^	159 (71)	157 (61)
Family’s past 2-year mean annual disposable income (1000 SEK)^a^	214 (84)	212 (102)
Net sick leave days in the previous year^a^	100 (125)	111 (135)
Disability pension^b^		
25%	61 (27)	54 (21)
50%	145 (65)	168 (66)
75%	17 (8)	34 (13)
Pain characteristics		
Duration (years)^a^	14.7 (8.9)	13.4 (9.4)
Numeric Rating Scale-10: past week pain intensity^a^	7 (2)	7 (1)
Primary pain location^b^		
Head	13 (6)	9 (4)
Neck, shoulders, and upper extremities	49 (22)	44 (17)
Upper back and chest	8 (4)	14 (5)
Lower back	38 (17)	33 (13)
Hips and lower extremities	20 (9)	32 (12)
Widespread or varying pain^c^	87 (39)	114 (45)
Most common primary ICD-10 diagnoses^b^,^d^		
Fibromyalgia (M79.7)	48 (22)	64 (25)
Myalgia (M79.1)	24 (11)	22 (9)
Unspecified pain (R52.9)	16 (7)	29 (11)
Weakness (R53.1)	19 (9)	16 (6)
Hospital and Anxiety Depression Scale: emotional distress^a^,^e^	46 (15)	47 (17)
Multidimensional Pain Inventory: interference^a^,	5 (1)	5 (1)
EuroQol-5-Dimensions: index^a,f^	0.2 (0.3)	0.2 (0.3)
Confidence in recovery^b^		
High	18 (8)	13 (5)
Moderate	33 (15)	24 (9)
Low	157 (70)	197 (77)
ICD-10 psychiatric comorbidity^b^,^d^	35 (16)	39 (15)
Schizophrenia spectrum disorder (F20-25, F28-29)	1 (0)	4 (2)
Bipolar disorder (F30-31)	9 (4)	9 (4)
Attention-deficit/hyperactivity disorder (F90)	13 (6)	10 (4)
Substance use disorder (F10-16, F18-19)	15 (7)	23 (9)
Self-harm (X60-64, Y10-34, Y87.0, Y87.2)	4 (2)	7 (3)

^a^mean (standard deviation).

^b^frequency (percent).

^c^pain in more than one of the locations that may vary over time.

^d^based on the International Statistical Classification of Diseases and Related Health Problems *10th* Revision (ICD-10).

^e^item response theory-based score scaled as percentiles according to procedure in supplementary materials of LoMartire et al. 2020.

^f^based on UK time trade-off.

**Fig 1:**
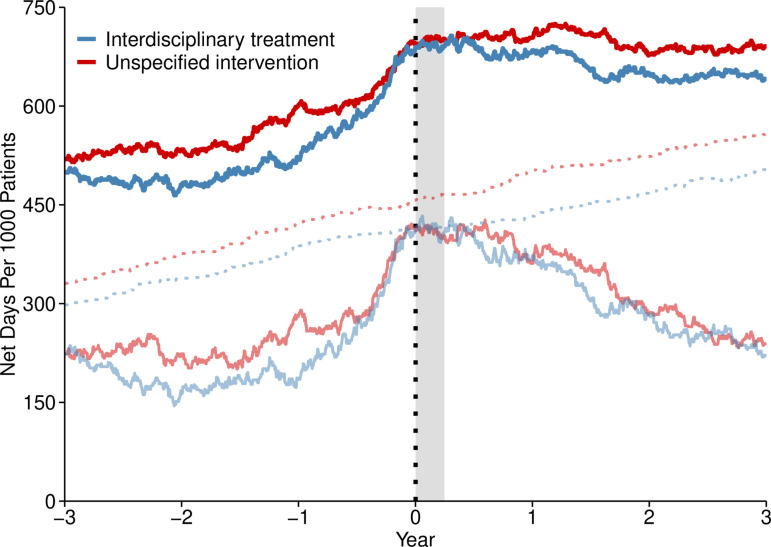
Mean net SA/DP days per 1000 patients with daily resolution. The thicker solid line represents SA/DP combined, whereas the thinner solid and dotted lines present sickness absence and disability pension, respectively. The dotted vertical line and the grey box denote specialist healthcare entry and the assumed IDT program period, respectively. Combined SA/DP computed with a daily cap per patient at 100%.

The descriptive overview suggested that IDT patients received less net SA/DP days than patients in unspecified interventions over the three years following specialist healthcare entry. As illustrated by Fig 1, the mean SA/DP days per 1000 patients were initially marginally lower in the IDT patients, equalized at the time of specialist healthcare entry, and then decreased marginally more for IDT patients towards the end of follow-up. Overall, IDT patients received a mean (standard deviation) of 729 (270) SA/DP days per patient compared to 766 (295) SA/DP days in other patients over the three-year period, which, on an annual basis, translated into 250 (93) *vs.* 257 (100), 243 (99) *vs.* 258 (106), and 235 (108) *vs.* 251 (109) for the first, second, and third year, respectively.

The analytical comparison, that was adjusted for the confounders in our conceptual model, supported that the amount of SA/DP days received over the three years following specialist healthcare entry was similar in both groups. In total, the mean (95% CI) SA/DP days were 749 (716, 781) for IDT patients compared to 739 (703, 774) for patients in unspecified interventions, which corresponded to a difference of 11 (-30, 51) SA/DP days. Moreover, there was no difference in the marginal risk of 1096 SA/DP days between intervention groups (risk difference [95% CI]: 0% [-8%, 5%]; risk ratio [95% CI]: 1.0 [0.6, 1.4]).

## Discussion

This study assessed whether IDT decreased SA/DP more than less intensive interventions in working age patients with chronic pain on partial disability pension. When comparing IDT with alternative treatments, we found no clinically meaningful difference between the two strategies.

There currently is no consensus about the effectiveness of IDT in reducing SA/DP for patients with chronic pain [6–8]. However, previous research indicates that a positive outcome of IDT is contingent on a poor SA/DP prognosis. In agreement, a randomized trial that compared the effects of IDT to alternative treatment strategies reported that a positive effect was limited to patients with a poor prognosis [13]. Concurrently, the prognosis of patients tends to be poorer in studies where IDT is favorable over other treatments than in studies where no difference is found. In support, studies focusing on patients with more extended SA at treatment start (e.g., > 6 months) [25–27], tend to find favorable effects with IDT, as opposed to patients with shorter term SA (e.g., <  3 months) [28–31]. In Swedish specialist healthcare, the criteria to enter into IDT overlaps significantly with the notion of poor prognosis and whereas time receiving SA/DP benefits certainly does not suffice as a sole criterion for establishing poor prognosis, it has been identified as a predictor of future SA/DP benefits in the general Swedish chronic pain population [20,32–35]. In light of the previous studies, it was reasonable to hypothesize that patients on partial disability pension would benefit from IDT, given their particularly poor prognosis. The absence of discernible differences between the two treatment types might be ascribed to a more intricate interplay of factors characterizing patients who derive additional benefits from IDT. Alternatively, it could be that existing IDT implementations simply do not lead to a reduction in SA/DP beyond what is achieved by alternative treatment strategies for working-age patients on partial disability pension. This highlights the need for improved patient selection procedures, potentially using machine-learning based algorithms that aid in decision support and more detailed information on IDT program characteristics [35,36].

Our results are based on high-quality register data from patients with chronic pain in Swedish specialist healthcare and generalizable to comparable patients in Sweden. Our two most important methodological limitations are that we assumed that the intervention groups were baseline equivalent after adjustment for our conceptual model and that the two contrasted interventions were sufficiently well-defined for consistent treatment effects. Whereas differences in unmeasured baseline confounders could have introduced some bias, groups were nearly identical in the measured baseline confounders and it is probably reasonable to assume that alternative treatment strategies generally implicate a lower level of involvement with the patient.

In summary, we compared the net SA/DP days over a three-year period between chronic pain patients in the working age on partial disability pension that were included in IDT programs or unspecified interventions at moderate-to-high volume specialist clinics. Our results suggest that IDT provides no advantage over less intensive interventions in decreasing SA/DP for this patient group, but we cannot rule out the possibility that it could be favorable for other groups of chronic pain patients with a poor prognosis.

## Supporting information

S1 Fig Patient sample flowchart. Flowchart of the sample selection procedure.
(PDF)

S2 Fig Directed acyclic graph of the conceptual model. The black arrow represents the effect of interest and the red arrows represent non-causal pathways due to confounding. Some arrows have been omitted to reduce clutter.
(PDF)

S3 Fig Distribution of the confounders adjusted for in the analysis (part 1). NA, missing data.
(PDF)

S4 Fig Distribution of the confounders adjusted for in the analysis (part 2). NA, missing data.
(PDF)

S5 Fig Outcome distribution per intervention group. Patients inside the grey square have total SA/DP days that are lower than the minimum expected days over the 3-year period under 25% DP. All patients were included in the analysis, but excluding patients in the grey square did not result in any meaningful changes to the results.
(PDF)

S6 Fig Total sickness absence and disability pension days by missingness status and intervention type. (PDF)

S7 Fig Bootstrap distribution based on ordinal logistic regression model. Figure shows the log(odds-ratio) of more SA/DP days given IDT. (A), the expected SA/DP days given IDT (B), the expected SA/DP days given unspecified intervention (C), and the expected difference in SA/DP days between IDT and unspecified intervention (D). Estimates are based on 10,000 bootstrap replicates. Dotted vertical lines mark 2.5th, 50th, and 97.5th percentiles.
(PDF)

S1 Table Covariate descriptives. (PDF)

S2 Table Ordinal logistic regression model of more SA/DP days. (PDF)

S3 Table Logistic regression model of 1096 SA/DP days. (PDF)
