# Supplementary figures and images for: The effect of interdisciplinary treatment on sickness absence and disability pension among chronic pain patients on partial disability pension
Source: PLoS One. 2025 Feb 4;20(2):e0317797. doi: 10.1371/journal.pone.0317797 (PMC11793736; doi:10.1371/journal.pone.0317797)

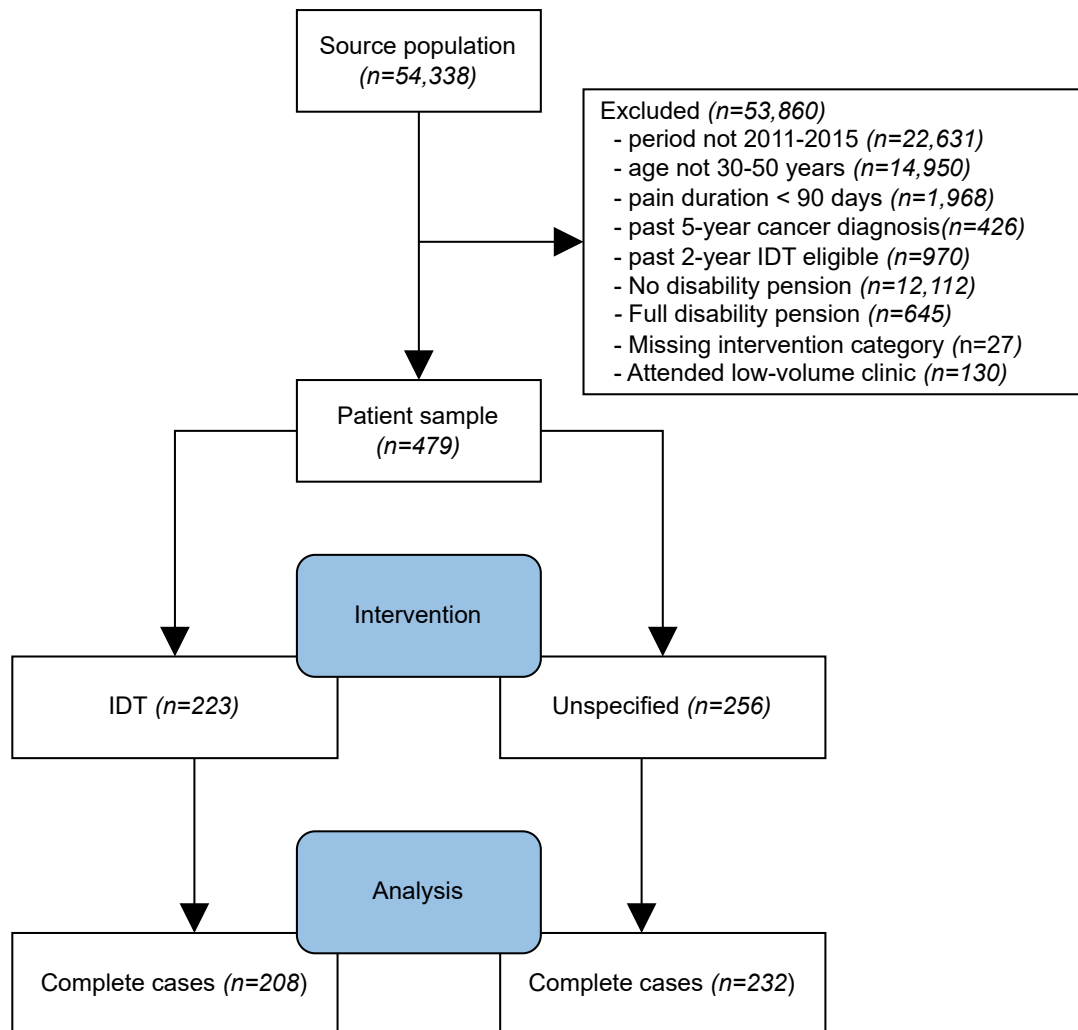

**S1 Figure:** Flow chart of the sample selection procedure.

Supplement: S1 Fig — Flowchart of the sample selection procedure. (PDF) [file pone.0317797.s001.pdf]

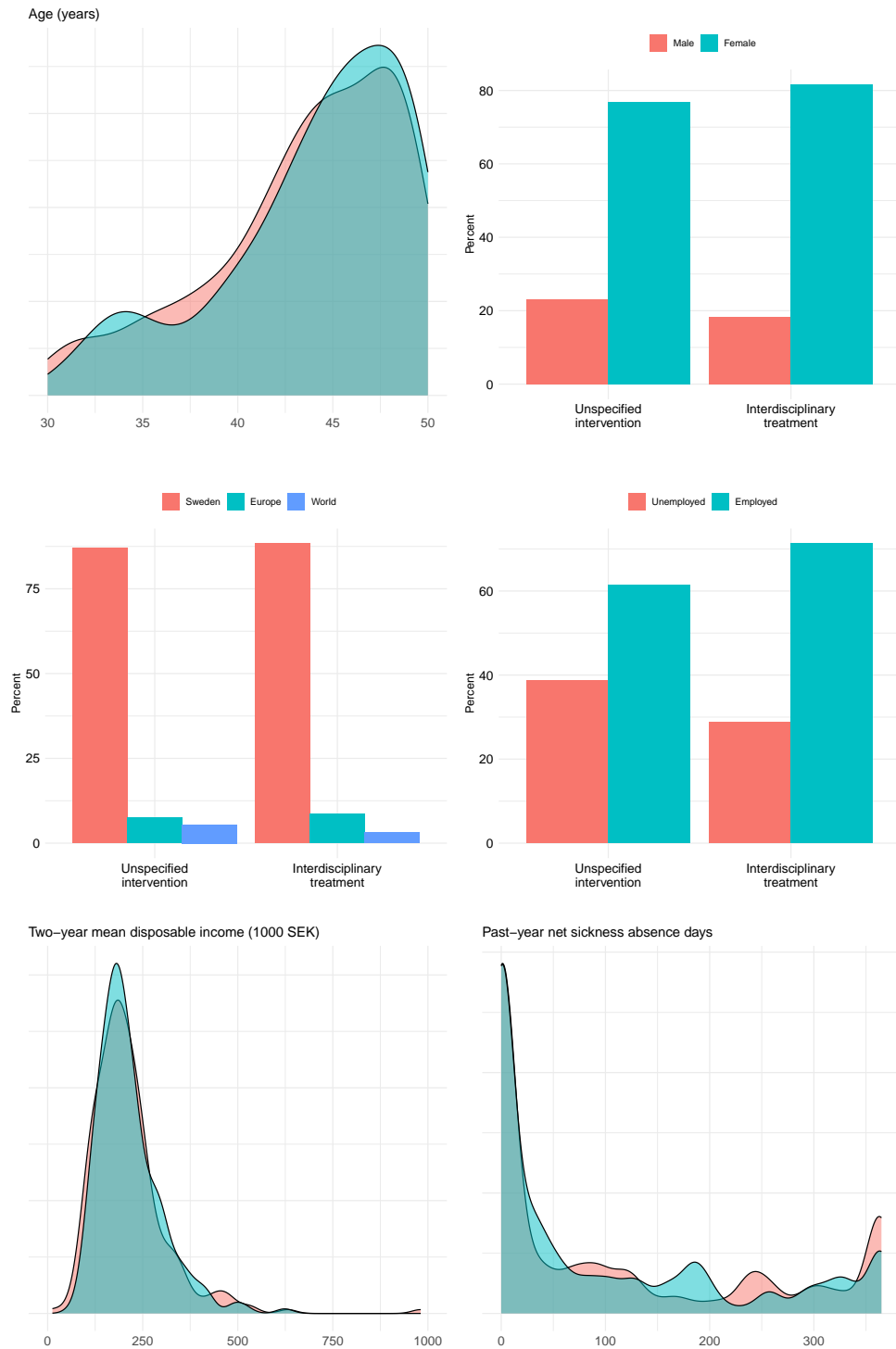

**S3 Figure: Distribution of the confounders adjusted for in the analysis (part 1). NA, missing data.**

Supplement: S3 Fig — NA, missing data. (PDF) [file pone.0317797.s003.pdf]

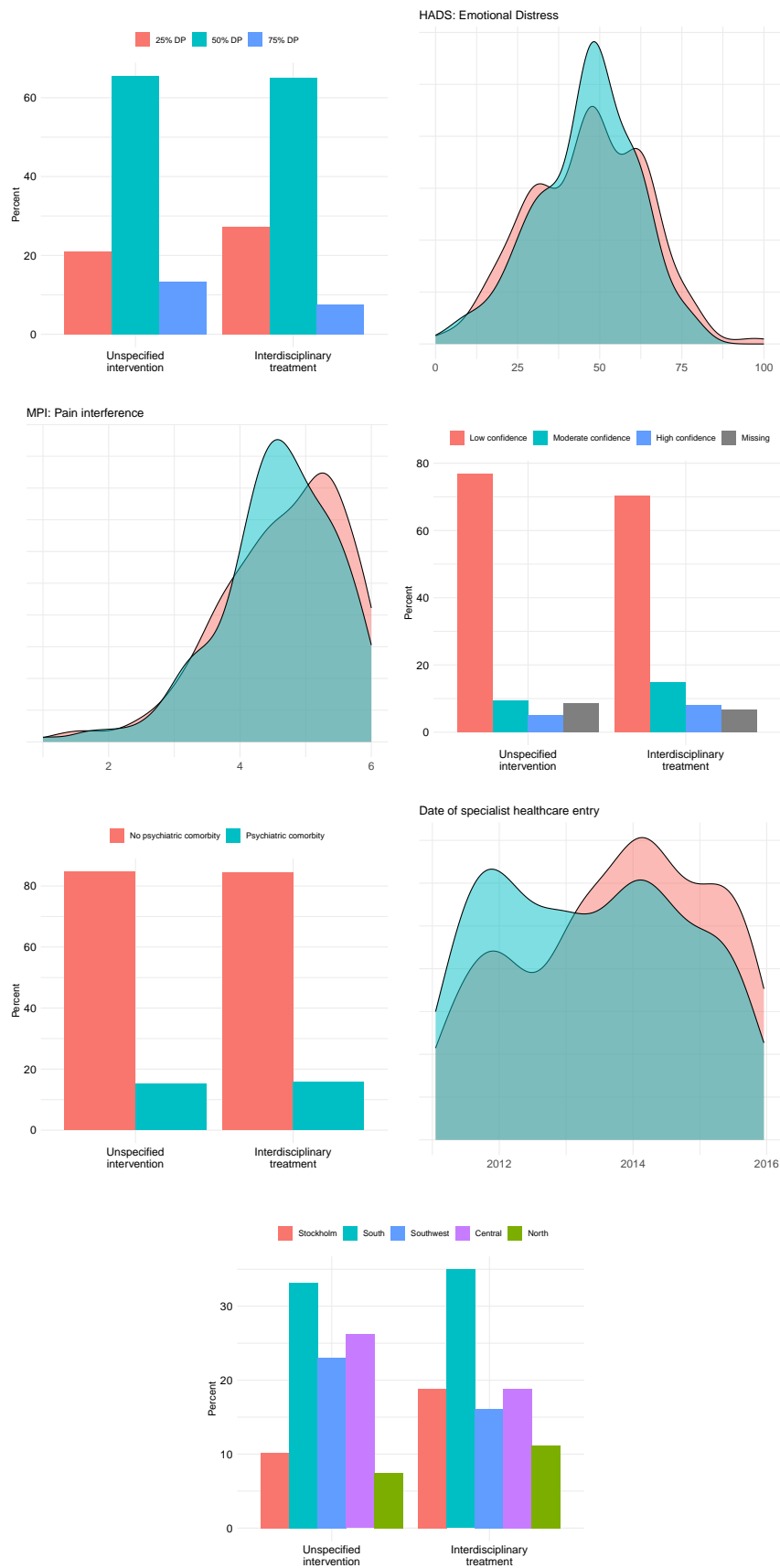

**S4 Figure: Distribution of the confounders adjusted for in the analysis (part 2).** NA, missing data.

Supplement: S4 Fig — NA, missing data. (PDF) [file pone.0317797.s004.pdf]
