# Supplementary material for: The effect of interdisciplinary treatment on sickness absence and disability pension among chronic pain patients on partial disability pension
Source: PLoS One. 2025 Feb 4;20(2):e0317797. doi: 10.1371/journal.pone.0317797 (PMC11793736; doi:10.1371/journal.pone.0317797)
Supplement: S2 Fig — The black arrow represents the effect of interest and the red arrows represent non-causal pathways due to confounding. Some arrows have been omitted to reduce clutter. (PDF) [file pone.0317797.s002.pdf]

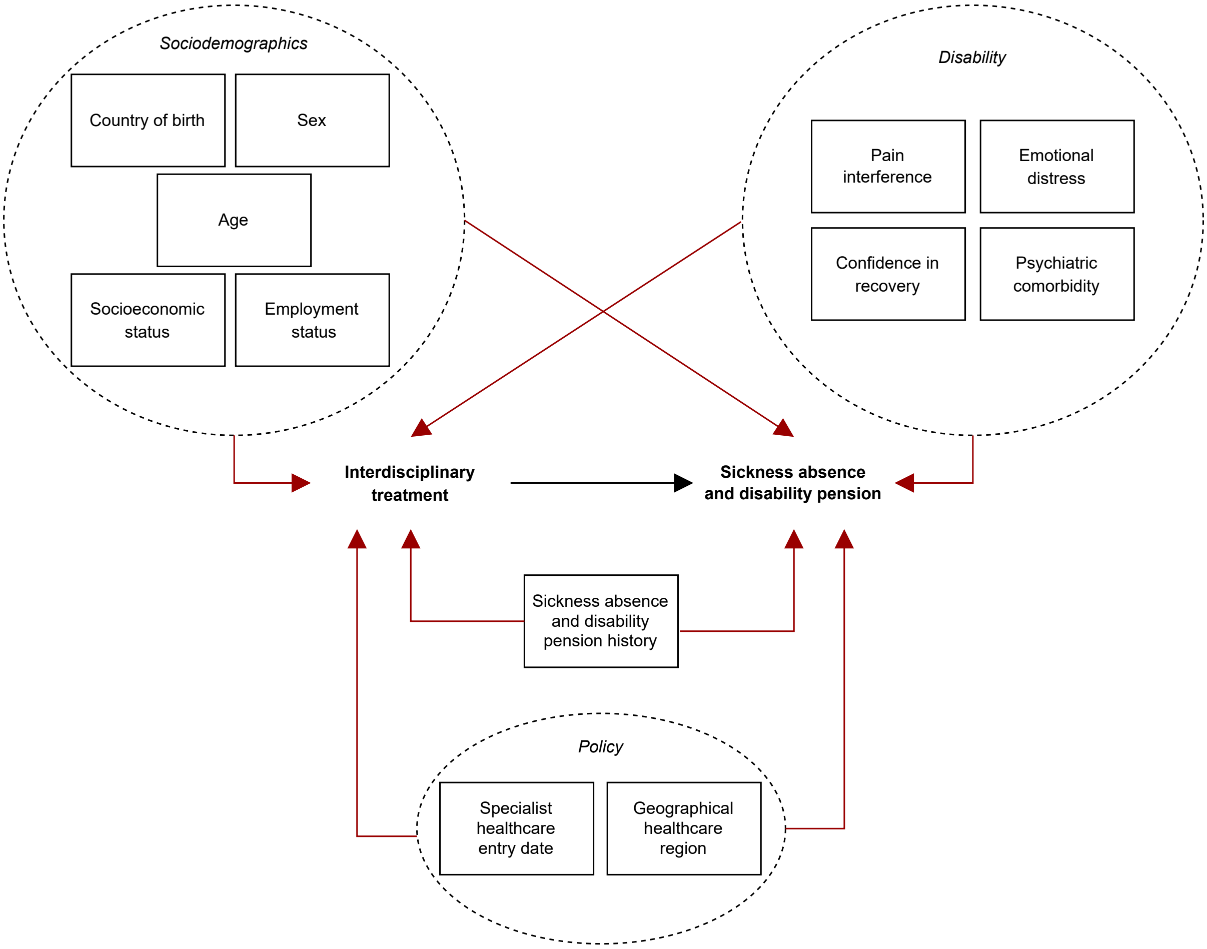

S2 Figure. Directed acyclic graph of the conceptual model. The black arrow represents the effect of interest and the red arrows noncausal pathways due to confounding. Some arrows have been omitted to reduce clutter.
