# Supplementary material for: The effect of interdisciplinary treatment on sickness absence and disability pension among chronic pain patients on partial disability pension
Source: PLoS One. 2025 Feb 4;20(2):e0317797. doi: 10.1371/journal.pone.0317797 (PMC11793736; doi:10.1371/journal.pone.0317797)
Supplement: S5 Fig — Patients inside the grey square have total SA/DP days that are lower than the minimum expected days over the 3-year period under 25% DP. All patients were included in the analysis, but excluding patients in the grey square did not result in any meaningful changes to the results. (PDF) [file pone.0317797.s005.pdf]

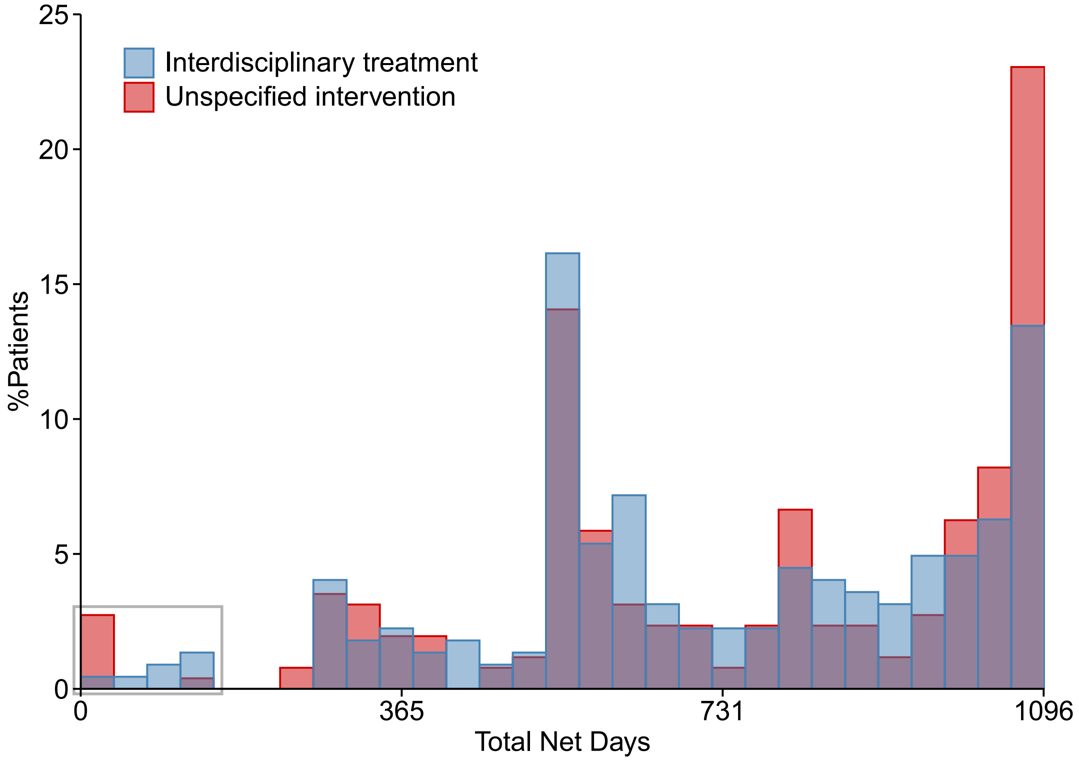

S5 Figure. Outcome distribution per intervention group. Patients inside the grey square have total SA/DP days that are lower than the minimum expected days over the 3-year period under 25% DP. All patients where included in the analys, but excluding patients in the gray square did not results in any meaningful changes to the results.
