# Supplementary material for: The effect of interdisciplinary treatment on sickness absence and disability pension among chronic pain patients on partial disability pension
Source: PLoS One. 2025 Feb 4;20(2):e0317797. doi: 10.1371/journal.pone.0317797 (PMC11793736; doi:10.1371/journal.pone.0317797)
Supplement: S6 Fig — (PDF) [file pone.0317797.s006.pdf]

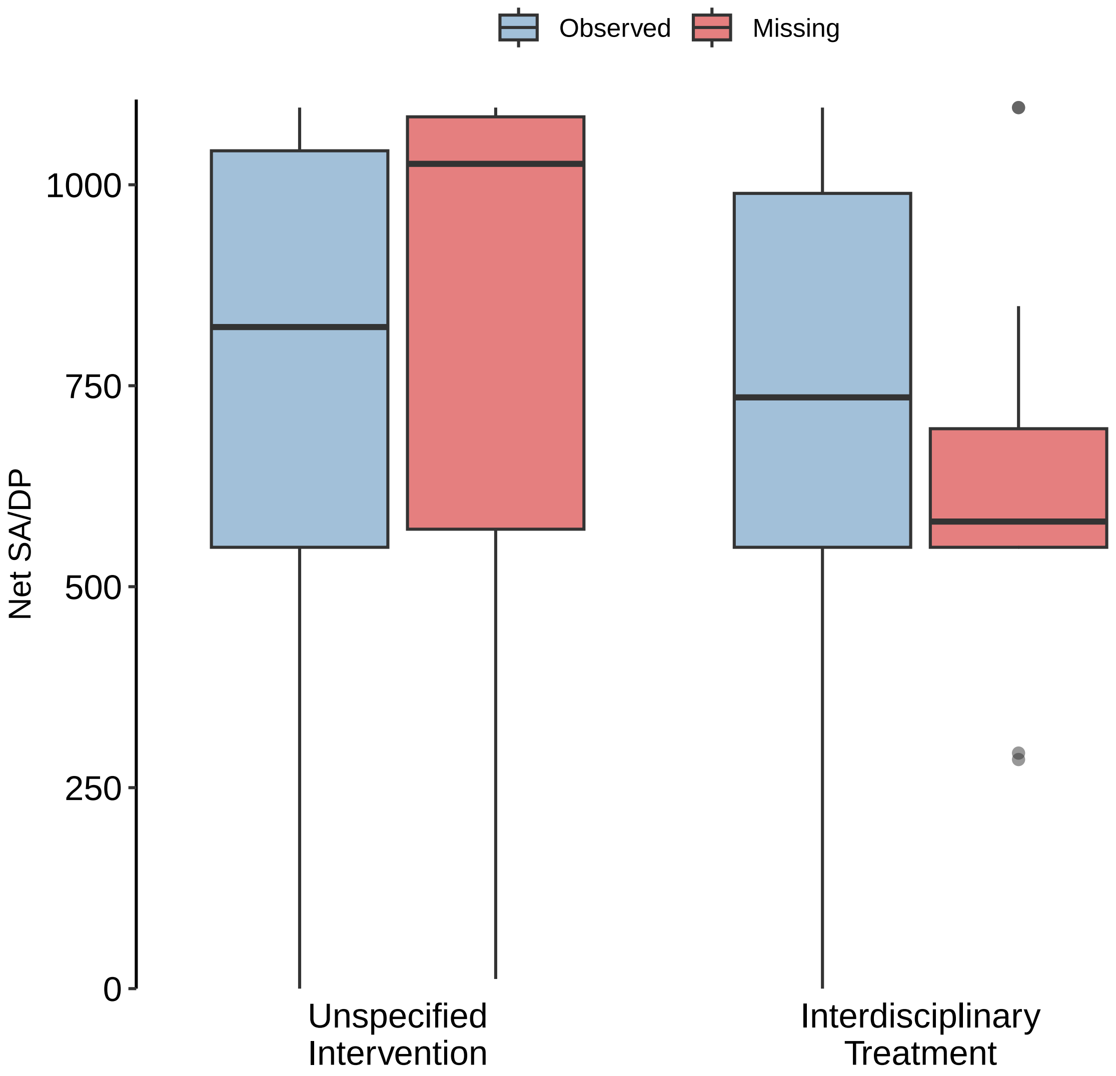

S6 Figure. Total sickness absence and disability pension days by missingness status and intervention type.
