# Supplementary material for: The effect of interdisciplinary treatment on sickness absence and disability pension among chronic pain patients on partial disability pension
Source: PLoS One. 2025 Feb 4;20(2):e0317797. doi: 10.1371/journal.pone.0317797 (PMC11793736; doi:10.1371/journal.pone.0317797)
Supplement: S7 Fig — Figure shows the log(odds-ratio) of more SA/DP days given IDT. (A), the expected SA/DP days given IDT (B), the expected SA/DP days given unspecified intervention (C), and the expected difference in SA/DP days between IDT and unspecified intervention (D). Estimates are based on 10,000 bootstrap replicates. Dotted vertical lines mark 2.5th, 50th, and 97.5th percentiles. (PDF) [file pone.0317797.s007.pdf]

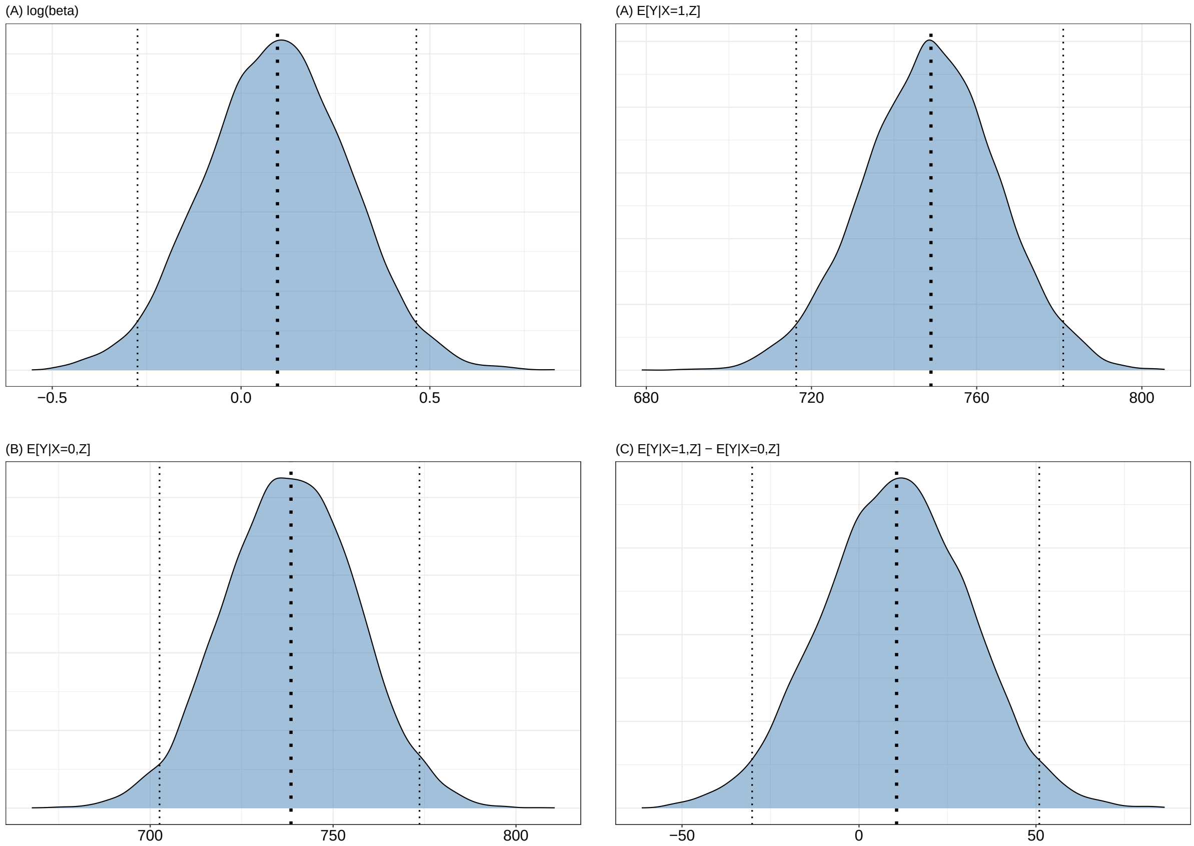

S7 Figure. Bootstrap distribution based on ordinal logistic regression model. Figure shows the log(odds-ratio) of more SA/DP days given IDT (A), the expected SA/DP days given IDT (B), the epected SA/DP days given unspecified intervention (C), and the expected dif-fernce in SA/DP days between IDT and unspecified intervention (D). Estimates are based on 10,000 bootstrap replicates. Dotted vertical lines mark 2.5th, 50th, and 97.5th percentiles.
