# Supplementary material for: The effect of interdisciplinary treatment on sickness absence and disability pension among chronic pain patients on partial disability pension
Source: PLoS One. 2025 Feb 4;20(2):e0317797. doi: 10.1371/journal.pone.0317797 (PMC11793736; doi:10.1371/journal.pone.0317797)
Supplement: S1 Table — (PDF) [file pone.0317797.s008.pdf]

S1 Table. Covariate descriptives.

Variable	Description	Data characteristics	Missing (n)	Source
Interdisciplinary treatment	IDT patient indicator	Binary indicator reported by care personnel	0	SQRP
Net sickness absence	Total days of net sickness absence in the year before start between 0-365 [0-1 scaled]	Based on date range and degree of episode from social insurance record	0	MiDAS
Disability pension status	Three categories of percent disability pension (25%, 50%, 75%)	Based on date range and degree from social insurance record	0	MiDAS
Age	Age between 30-50 years [0-1 scaled]	Based on month and year of birth from national record	0	LISA
Sex	Female indicator	Binary indicator from national record	0	LISA
Birth region	Sweden; Europe excl Sweden; Outside Europe	Three categories from national record	0	LISA
Employed	Employment status indicator	Binary indicator from social insurance record and patient self-report data	0	MiDAS/ SQRP
Disposable income	Two-year mean of the family's disposable income in KSEK [min-max scaled]	Based on annual disposable income from national record	0	LISA
Emotional distress	HADS IRT score [0-1 scaled]	Patient self-report questionnaire data	0	SQRP
High pain interference	Indicator for MPI pain severity scale > 4 points	Binary indicator based on patient self-report questionnaire data	11	SQRP
Confidence in recovery	Low; moderate; high	Three categories based on patient self-report questionnaire data	37	SQRP
Psychiatric comorbidity	Indicator for ICD-10: F20-25; F28-29; F30-31; F90; F10-16; F18-19; X60-64; Y10-34; Y87.0; Y87.2	Binary indicator based on patient journal record aggregated in NPR	0	NPR
Entry year	Specialist healthcare entry year between 2011-2015	Five categories based on date of first visit reported by care personnel	0	SQRP
Healthcare region	Stockholm/Gotland; Southern Sweden; Soutwestern Sweden; Central Sweden; Northen Sweden	Five categories based on clinic's location	0	SQRP

SQRP, Swedish Quality Register for Pain Rehabilitation. MiDAS, Micro Data for Analysis of the Social Insurance register. LISA, Longitudinal Integration Database for Health Insurance and Labour Market Studies. NPR, National Patient Register.
