# Supplementary material for: The effect of interdisciplinary treatment on sickness absence and disability pension among chronic pain patients on partial disability pension
Source: PLoS One. 2025 Feb 4;20(2):e0317797. doi: 10.1371/journal.pone.0317797 (PMC11793736; doi:10.1371/journal.pone.0317797)
Supplement: S2 Table — (PDF) [file pone.0317797.s009.pdf]

S2 Table. Ordinal logistic regression model of more SA/DP days

Model term	Est (95% CI)	Std Error	P-value
Interdisciplinary treatment	0.09 (-0.25, 0.44)	0.18	0.603
Net sickness absence	3.43 (2.85, 4.02)	0.30	0.000
50% Disability pension	1.74 (1.30, 2.19)	0.23	0.000
75% Disability pension	2.85 (2.18, 3.52)	0.34	0.000
Age	1.32 (0.23, 2.41)	0.56	0.018
Female	-0.20 (-0.63, 0.22)	0.22	0.349
Born in Europe	0.65 (0.00, 1.30)	0.33	0.051
Born outside Europe	0.25 (-0.59, 1.10)	0.43	0.558
Employed	-0.18 (-0.57, 0.22)	0.20	0.378
Disposable income	-0.13 (-1.94, 1.67)	0.92	0.885
Emotional distress	0.17 (-0.97, 1.30)	0.58	0.774
High pain interference	0.33 (-0.09, 0.75)	0.22	0.127
Moderate confidence in recovery	-0.25 (-0.76, 0.27)	0.26	0.348
High confidence in recovery	0.08 (-0.61, 0.78)	0.36	0.813
Psychiatric comorbitity	0.34 (-0.13, 0.81)	0.24	0.160
Specialist healthcare entry year 2012	-0.04 (-0.60, 0.51)	0.28	0.884
Specialist healthcare entry year 2013	0.19 (-0.32, 0.70)	0.26	0.472
Specialist healthcare entry year 2014	0.56 (0.03, 1.09)	0.27	0.039
Specialist healthcare entry year 2015	0.67 (0.12, 1.22)	0.28	0.017
Southern Sweden	0.74 (0.21, 1.26)	0.27	0.006
Southwestern Sweden	0.41 (-0.18, 0.99)	0.30	0.173
Central Sweden	0.37 (-0.21, 0.94)	0.29	0.209
Northern Sweden	1.04 (0.33, 1.75)	0.36	0.004
