# Supplementary material for: The effect of interdisciplinary treatment on sickness absence and disability pension among chronic pain patients on partial disability pension
Source: PLoS One. 2025 Feb 4;20(2):e0317797. doi: 10.1371/journal.pone.0317797 (PMC11793736; doi:10.1371/journal.pone.0317797)
Supplement: S3 Table — (PDF) [file pone.0317797.s010.pdf]

S Table. Logistic regression model of 1096 SA/DP days

Model term	Est (95% CI)	Std Error	P-value
(Intercept)	-8.96 (-13.44, -4.95)	2.16	0.000
Interdisciplinary treatment	0.04 (-0.68, 0.75)	0.36	0.922
Net sickness absence	3.11 (2.14, 4.19)	0.52	0.000
50% Disability pension	1.91 (0.90, 3.08)	0.55	0.001
75% Disability pension	0.60 (-1.24, 2.29)	0.88	0.496
Age	-0.41 (-2.55, 1.81)	1.11	0.712
Female	-0.24 (-1.12, 0.69)	0.46	0.600
Born in Europe	1.09 (-0.29, 2.39)	0.68	0.107
Born outside Europe	-0.71 (-3.73, 1.24)	1.15	0.536
Employed	-0.33 (-1.09, 0.43)	0.39	0.390
Disposable income	2.39 (-0.94, 5.54)	1.63	0.141
Emotional distress	-0.19 (-2.55, 2.19)	1.20	0.876
High pain interference	2.16 (0.83, 3.78)	0.74	0.004
Moderate confidence in recovery	-0.69 (-1.97, 0.42)	0.60	0.250
High confidence in recovery	0.94 (-0.52, 2.32)	0.72	0.188
Psychiatric comorbitity	0.14 (-0.91, 1.14)	0.52	0.783
Specialist healthcare entry year 2012	0.04 (-1.66, 1.74)	0.85	0.963
Specialist healthcare entry year 2013	0.76 (-0.62, 2.28)	0.73	0.297
Specialist healthcare entry year 2014	2.14 (0.87, 3.63)	0.69	0.002
Specialist healthcare entry year 2015	2.67 (1.41, 4.16)	0.69	0.000
Southern Sweden	1.03 (-0.36, 2.71)	0.77	0.177
Southwestern Sweden	1.07 (-0.49, 2.86)	0.84	0.201
Central Sweden	1.78 (0.34, 3.51)	0.79	0.025
Northern Sweden	2.44 (0.83, 4.29)	0.87	0.005
